# MIDPERIPHERAL RETINAL THICKENING ON WIDEFIELD OPTICAL COHERENCE TOMOGRAPHY IN A PATIENT WITH A MUTATION IN THE *NR2E3* GENE

**DOI:** 10.1097/ICB.0000000000001615

**Published:** 2024-06-11

**Authors:** Jan Willem R. Pott, E. Angela Huiskamp, Joke B. G. M. Verheij

**Affiliations:** Departments of * Ophthalmology, and; † Genetics, University Medical Center Groningen, University of Groningen, Groningen, the Netherlands.

**Keywords:** enhanced S-cone syndrome, NR2E3, widefield OCT

## Abstract

**Purpose:**

To report typical retinal findings in a girl with the presumed diagnosis of enhanced S-cone syndrome, attributed to the presence of a homozygous likely pathogenic variation in the *NR2E3* gene.

**Methods:**

A retrospective case report with multimodal imaging studies.

**Results:**

Widefield OCT examination at the age of 11 showed increased thickening of the midperipheral retina. In the area of retinal thickening, the delineation of retinal architecture was coarse and photoreceptor outer segments thinned and disrupted. The retinal pigment epithelium appeared to be normal on autofluorescence imaging. In retrospect, the retinal thickening was already apparent on conventional OCT at the age of 3.

**Conclusion:**

Widefield OCT is a valuable new tool in assessing typical retinal abnormalities in patients with NR2E3 mutations, by showing an abnormal but very characteristic thickening of the midperipheral retina.

## Abstract 2

This study describes a typical midperipheral retinal thickening with coarse delineation on widefield OCT in an 11-year-old girl with the presumed diagnosis of enhanced S-cone syndrome (ESCS), attributed to the presence of a homozygous likely pathogenic variation in the *NR2E3* gene. This retinal finding has been described in earlier studies of patients with ESCS, by scanning off-center with conventional OCT examination.

Mutations in NR2R3 are associated with Goldmann–Favre syndrome, also known as enhanced S-cone syndrome (ESCS), and some forms of dominant and autosomal recessive inherited retinitis pigmentosa. This gene has a role in retinal photoreceptor differentiation during retinogenesis, regulating the fate of retinal progenitor cells. In ESCS, dysfunction of the NR2E3 gene will lead to loss of rod function and an excess of S-cones (short-wavelength) at the expense of M-cones (medium-wavelength) and L-cones (long-wavelength).^1,2^ The earliest symptom of ECSC is nyctalopia, and in some patients, a decreased visual acuity and/or nystagmus is seen. Diagnosing ESCS is difficult, as visible retinal abnormalities vary in patients or may be absent in younger patients. Frequently described abnormalities are as follows: yellow-white lesions in the posterior pole, nummular and clumped pigmentation at the level of RPE around the vascular arches, and foveomacular schisis. Other abnormalities are submacular fibrosis and torpedo-like atrophic lesions. Diagnosis of ESCS should be confirmed by electrophysiologic testing, as patients have pathognomonic electroretinogram (ERG) responses. However, ERG testing requires specialized protocols, not always available in every clinic.^2–4^ We describe a young girl with presumed ESCS, but lacking electrophysiological confirmation of this diagnosis. The peripheral retina showed a very typical thickening of the retina outside the macular zone, with loss of lamination, frequently described in ESCS. This aspect of the retina can be easily overlooked in conventional central OCT imaging. However, it is easily shown on widefield swept source OCT imaging of the retina.

## Case Report

**Fig. 1.**
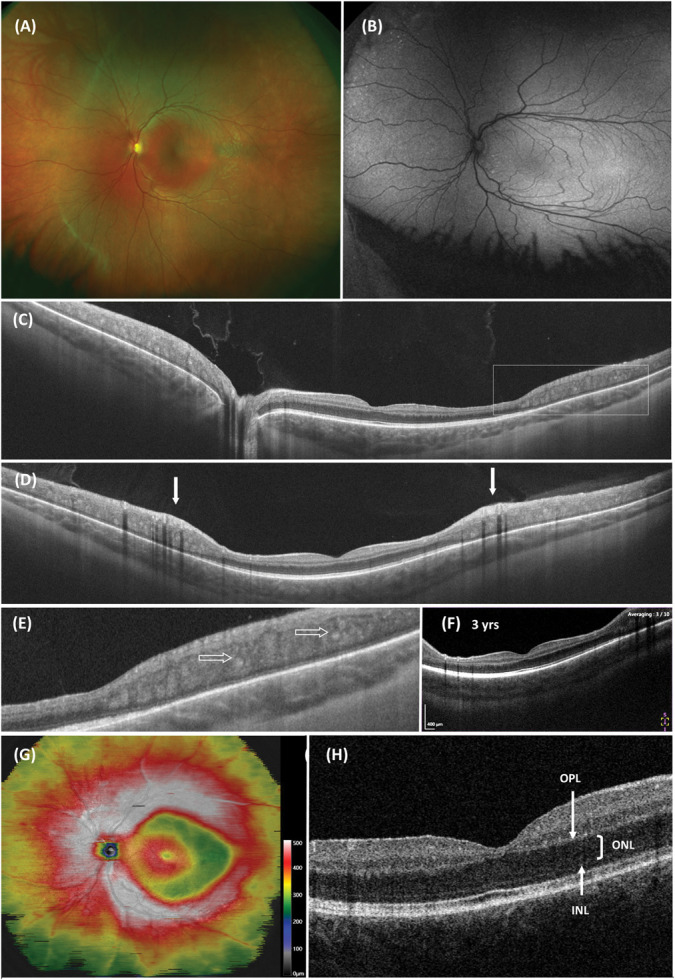
All presented imaging originates from examinations conducted at the age of 11, with the exception of capture F, which was obtained when the patient was 3 years of age. **A.** Ultra-widefield retinal image of the left eye (Optos, CA). **B.** Autofluorescence image of the left eye made with ultra-widefield fundus camera (Optos CA). There is a slight distortion of the image due to the nystagmus. Note a few hyperreflective spots between the optic nerve and macula and in the far nasal peripheral retina. Otherwise, there is no abnormal reflex, especially not in the area around the vascular arches. **C** and **D.** Horizontal and vertical widefield swept source OCT of the retina of the left eye (Canon Xephilio OCT-S1). Note the sudden increase in retinal thickness just outside the macular area. The vascular arches are indicated by white arrows. **E.** Magnified detail (insert from (**C**) indicated by box) of midperipheral retina, with an increase in thickness and loss of delineation and with apparent hyperreflective dots (open arrows). Normal organization of photoreceptors is lost with the absence of myoid zone, ellipsoid zone, and outer segments of photoreceptors. **F.** Regular vertical OCT scan (Canon, OCT HS100) through the macular region made at 3 years of age. Note the increase in retinal thickness at roughly the same anatomical border as in the recent widefield OCT image (**D**). **G.** Retinal thickness map of the left eye made with widefield OCT scan. Note the sharp increase in retinal thickness just inside the vascular arch and following this contour. Retinal thickness decreases in a more gradual pattern toward the far periphery of the retina. Only left-eye imaging is shown here. The OCT of the right eye gave similar results. **H.** OCT scan of the central macular area. Note the increased thickness of ONL. No foveal hypoplasia was present at all ages. The macular scans in C, D, and F are slightly off-center.

We present an 11-year-old girl with a presumed diagnosis of ESCS, who presented at our clinic at the age of four months, with a nystagmus and abnormal visual responses. On fundoscopy, she had no pigmentary alterations. An electroretinogram at the age of 1 showed only a weak response to 30-Hz flicker stimuli. Detection of other responses under scotopic or photopic conditions was not possible due to the increased rate of background noise. At 2 years, genetic testing revealed a homozygous likely pathogenic variation in the NR2E3 gene (Chr15(GRCh37): g.72109962_72109963del; NM_014249.2:c.1171_1172del (p.(Phe391 fs)). The variant was validated by Sanger sequencing, and it induces a frameshift and a premature stop in the last exon of the NR2E3 gene. There is likely no nonsense-mediated decay of the mRNA, but rather, a shorter protein is produced, lacking the final six amino acids. The loss of this portion of the protein may potentially impact the ligand binding of the photoreceptor-specific nuclear receptor. This mutation has previously been documented in the literature as being pathogenic.^5^ This variant is suggestive for the diagnosis of ESCS, although this diagnosis should normally be confirmed by ERG. However, the parents refused further electrophysiological testing, even at a later age. At 3 years, ultra-widefield fundus imaging and OCT scans of the macula were made: short-wavelength autofluorescence imaging showed some hyperautofluorescent lesions alongside the vascular arch and in the peripheral retina. Macular OCT scans showed the absence of foveal retinoschisis and normal delineation of the central retina. Ophthalmologic examination remained stable over the years. Binocular visual acuity at 11 years of age was 0.4, and the child still had horizontal nystagmus with compensatory ocular torticollis. Refraction was stable with a characteristic hypermetropia for ESCS of S+6,00 C-1,50 x175 and S+6,75 C-2,00 x175 of the right and left eye. Recently, at the age of 11, we were able to make a widefield OCT showing a typical highly increased thickness of the midperipheral retina in both eyes, starting just inside the vascular arches and extending to the further periphery (see Figure 1). The midperipheral retinal thickness was highest at the location inferior to the macula with a thickness of 510 *μ*m in the right eye and 515 *μ*m in the left eye (the location of measurement is roughly indicated by the right white arrow in Figure 1D). In this zone with highly increased retinal thickness was coarse, photoreceptor inner and outer segments thinned to absent and disrupted, but RPE appeared to be normal on autofluorescence imaging.

In retrospect, the increased thickness of the midperipheral retina was already apparent in the earliest vertical OCT scans, at the age of 3, implying a stable localization of this feature.

**Fig. 2.**
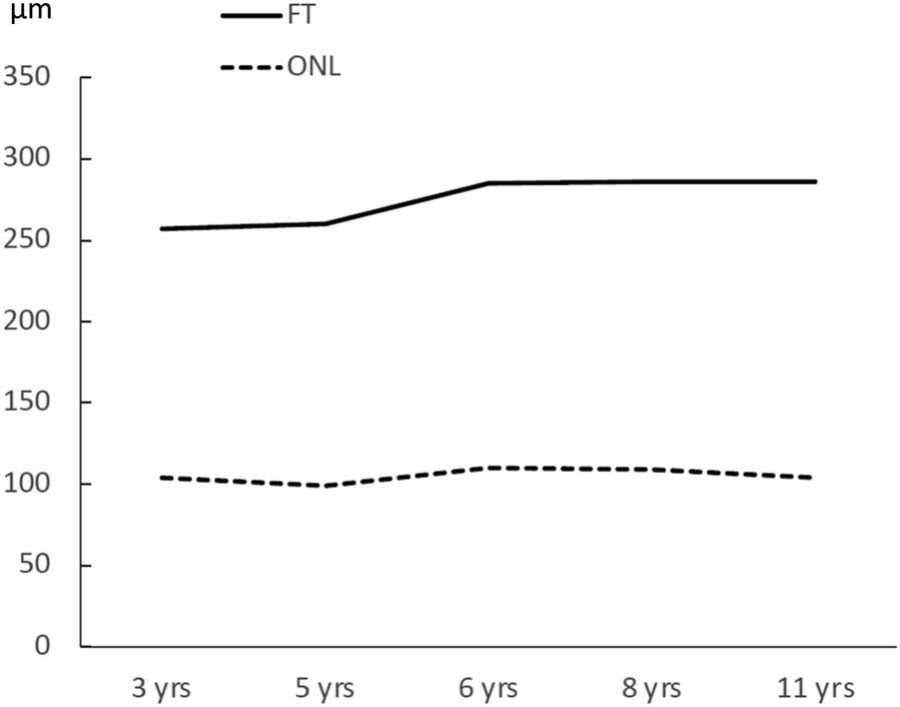
Follow-up of foveal thickness (FVT), and thickness of the outer nuclear layer (ONL) of the right eye from the age of 3 years to 11 years as measured with the OCT scan. The vertical axes represent the thickness expressed in micrometers (μm), while the horizontal axes represent the age of the patient.

The central retina of both eyes showed a rather normal retinal lamination. Nevertheless, there was an increase in overall central retinal thickness. At the age of 11, thickness at the foveal location was 286 *µ*m in the right eye and 290 *µ*m in the left eye, which was above the intrinsic normative values of the OCT scan. For comparison, in a recent large study on foveal thickness measured in a normal population, the mean value within the age 10-19 years was 232.1 *µ*m (SD ± 18.35 *µ*m, with a 95% confidence interval (CI) of 226.3 *µ*m–237.7 *µ*m).^6^ The outer nuclear layer (ONL) in our patient had a more apparent increased thickness of 104 *µ*m in the right eye and 103 *µ*m in the left eye, when measured inferior to the fovea, at the location of highest parafoveal thickness. For comparison, in a recent study of normative data for retinal lamination in children with a mean age of 12 years, the values for the ONL in this area were 69.4 *µ*m (SD ±7.72 *µ*m, with a 95% CI of 68.04 *µ*m and 70.77 *µ*m).^7^ We obtained reasonable OCT scans of the central retinal area of the right eye from the age of 3 years to 11 years, allowing us to measure both foveal thickness and the thickness of the ONL longitudinally. The ONL thickness was consistently measured at the same location during each examination. Retinal lamination was manually adjusted whenever required. Figure 2 illustrates the results of foveal thickness and ONL thickness measurements throughout the follow-up period. Foveal thickness increased minimally during follow-up, and ONL thickness was stable throughout the years.

## Discussion

In this case report, we illustrate typical retinal image findings with hyperthickness and coarse lamination in all quadrants of the midperipheral retina, in a girl exhibiting signs suggestive of ESCS, attributed to the presence of a disease-causing variant in the *NR2E3* gene. The patient initially presented as an infant with symptoms including nystagmus, abnormal visual responses, and a markedly reduced ERG, with only discernible weak responses at 30-Hz flicker stimulation. Considering the potential variability in ERG recordings at one year of age, it is our standard practice to repeat such assessments. Unfortunately, a subsequent ERG assessment at a later age was not feasible, preventing definitive confirmation of the ESCS diagnosis. At the age of 11, the patient had not yet manifested the characteristic retinal pigmentation commonly associated with ESCS. Typically, individuals with ESCS present in the second decade of life reporting nyctalopia, accompanied by distinct macular abnormalities and peripheral (pigmentary) retinal lesions. A presentation of ESCS at a very young age with decreased visual responses and nystagmus is uncommon, as highlighted in the recent case series of De Carvalho et al.^2^ Jacobson et al.^8^ were the first to mention the increase in midperipheral retinal thickening in ESCS. In their study of 17 patients with NR2E3 mutations, all patients showed a marked increase in the thickness of the retina beyond the central retina. They postulated that the thickening and loss of lamination in ESCS is due to a failure of embryonal rod photoreceptor differentiation, an increase in the number of larger cells with S-cone phenotype, and the continued proliferation of retinal glial cells. Furthermore, they reported an increase in the ONL of the central retina with preserved lamination. Ammar et al^9^ recently reported a case of a 13-year-old boy with ESCS, having a similar widefield OCT (16.4 mm) appearance with increased thickness and loss of lamination of the nasal peripheral retina. Other studies with higher resolution OCT scans also mentioned the coarse lamination and disorganization in the midperipheral retina of patients with ESCS when scanning off-center. On the other hand, these studies did not report the typical increased thickness of the midperipheral retina.^2^ This abnormal region may fall just beyond the scope of conventional OCT scans. The (ultra) widefield OCT scan offers the benefit of capturing both the central and peripheral retina within a single image, thereby highlighting any increased thickness of the midperipheral retina.

Reliable OCT scans in the present case were performed over a period of 8 years, from the age of 3 years until 11 years. During this period, the border of the retina with normal delineation to the retina with increased thickness and loss of lamination remained relatively consistent in its location. In addition, the central retina's anatomical organization showed no significant alterations, with the ONL maintaining a consistent thickness over these years. This observation has significance in the context of emerging therapies for inherited retinal disorders originating from mutations in the *NR2E3* gene.

In conclusion, we think that the widefield OCT may be a valuable new tool in assessing typical retinal abnormalities of ESCS, by showing an abnormal but very characteristic hyperthickening of the midperipheral retina with loss of lamination. Studies with a larger number of patients with ESCS examined with widefield OCT are needed to confirm the validity of this single observation.
